# β-Asarone Induces Apoptosis and Cell Cycle Arrest of Human Glioma U251 Cells via Suppression of HnRNP A2/B1-Mediated Pathway In Vitro and In Vivo

**DOI:** 10.3390/molecules23051072

**Published:** 2018-05-03

**Authors:** Li Li, Yi Yang, Mingxia Wu, Zanyang Yu, Chengqiang Wang, Guojun Dou, Hui He, Hongmei Wang, Na Yang, Hongyi Qi, Xiaoyu Xu

**Affiliations:** 1 College of Pharmaceutical Sciences, Southwest University, Chongqing 400716, China; lilyli@email.swu.edu.cn (L.L.); doria11@email.swu.edu.cn (Y.Y.); zhangww_2005@126.com (M.W.); y1128y@email.swu.edu.cn (Z.Y.); wangchengqiang432@163.com (C.W.); guojundou@163.com (G.D.); hehui662299@163.com (H.H.); wanglitcm@sina.com (H.W.); 2 Institute of Laboratory Animal Sciences, Sichuan Academy of Medical Sciences and Sichuan Provincial People’s Hospital, Chengdu 610212, China; nanayang@yeah.net

**Keywords:** β-asarone, glioma, apoptosis, cell cycle arrest, hnRNPA2/B1, alternative splicing

## Abstract

HnRNP A2/B1 has been found to be an oncogenic protein strongly related to the growth of human glioma cells. Herein, β-asarone, the main component in the volatile oil of *Acori tatarinowii* Rhizoma, inhibited the cell viability, proliferation, and colony formation ability of U251 cells. Moreover, β-asarone induced apoptosis and cell cycle arrest at the G1 phase. Notably, β-asarone suppressed the expression of hnRNP A2/B1 and hnRNPA2/B1 overexpression remarkably reversed β-asarone-mediated apoptosis and cell cycle arrest. Importantly, β-asarone promoted the alternative splicing of Bcl-x by enhancing the ratio of Bcl-xS/Bcl-xL. Meanwhile, hnRNPA2/B1 overexpression mitigated the promoting effect of β-asarone on the alternative splicing of Bcl-x. β-asarone also regulated the level of the key proteins involved in the death receptor pathway and mitochondrial apoptosis pathway. Additionally, β-asarone modulated the cell cycle-related proteins p21, p27, Cdc25A, cyclin D, cyclin E, and CDK2. Finally, β-asarone inhibited tumor growth and induced apoptosis in nude mice bearing U251 tumor xenografts. β-asarone also suppressed the hnRNP A2/B1 expression, enhanced the expression of cleaved-caspase 3 and p27 and the ratio of Bcl-xS/Bcl-xL, and reduced the expression of CDK2 in U251 xenografts. Together, β-asarone-induced apoptosis and cell cycle arrest of U251 cells may be related to the suppression of hnRNPA2/B1-mediated signaling pathway.

## 1. Introduction

Glioma derived from the neural ectoderm is the most common brain tumor and characterized by rapid growth, high invasiveness, and a poor prognosis [1]. In the United States, brain and other nervous system tumors have been described as the leading cause of cancer death for men before age 40 years and for women before 20 years. The number of deaths from brain and other nervous system tumors was estimated to be 16,830 in 2018, which represents a slight increase compared to that in 2017 for both men and women [2]. Currently, the clinical efficacy of conventional chemotherapy is extremely restricted due to the blood–brain barrier (BBB), serious side effects, and multidrug resistance [3]. Thus, new alternative treatment strategies are urgently needed for malignant glioma therapy.

Heterogeneous nuclear ribonucleoproteins (hnRNPs) make up a large family of RNA-binding proteins associating with nascent pre-mRNA transcripts in eukaryotic cells and play diverse roles in RNA processing, pre-mRNA splicing, mRNA export, localization, translation, and stability [4]. HnRNP A2/B1, including hnRNP A2 and its splicing variant hnRNP B1, encoded by the *HNRNPA2/B1* gene, is among the most abundant hnRNP proteins [5]. Accumulating evidence has demonstrated that hnRNP A2/B1 is oncogenic and overexpressed in various tumor cells, including breast [6], pancreas [7], liver [8], gastric [9], and lung carcinoma cells [10]. Moreover, hnRNP A2/B1 overexpression has also been observed in human glioma tissue specimens and is closely correlated with advanced glioma grades [5,11]. It is becoming increasingly clear that deregulation of alternative splicing involved in processing pre-mRNAs of diverse signaling proteins plays a direct role in cancer development and progression [12]. Recently, hnRNP A2/B1 has been described in the regulation of alternative splicing of several tumor suppressors and oncogenes, such as Bcl-x [13,14,15], which is an anti-apoptotic protein belonging to the well-known Bcl-2 family. Moreover, accumulating evidence also revealed that suppression of hnRNP A2/B1 induced cell cycle arrest at G1 phase in cervical cancer cells [16], lung cancer cells [17,18], and human embryonic stem cells [19], which renders it a potential novel target for tumor therapy.

β-asarone is the main component in the volatile oil of *Acori tatarinowii* Rhizoma, a Chinese herbal medicine proved to possess anti-glioma activity in our recent study [20]. It has been described that β-asarone exhibited anti-tumor activities on colorectal cancer cells [21,22] and gastric cancer cells [23]. Recently, we found that β-asarone obviously inhibited the growth of glioma cells [24], which was further confirmed by another group [25]. Moreover, β-asarone has been shown to not only directly cross the blood–brain barrier (BBB), but also to improve the permeability of the BBB and inhibit the function of P-glycoprotein [26,27,28]. A two-dimensional gel electrophoresis-based proteomics has been recently employed by our group to comprehensively investigate the cellular targets of β-asarone. HnRNP A2/B1 was successfully identified as one of the key protein targets regulated by β-asarone [24]. Most recently, we found that β-asarone inhibited invasion and the epithelial–mesenchymal transition (EMT) in U251 cells by suppressing HnRNP A2/B1 [29]. Thus, it is interesting for us to further explore the potential role of hnRNP A2/B1-mediated signaling pathway in the anti-glioma effect of β-asarone.

In the current study, we further characterized the inhibitory effect of β-asarone on the growth of U251 cells. Then, the induction of apoptosis and cell cycle arrest by β-asarone was determined. Furthermore, we also sought to identify the underlying role of hnRNP A2/B1 and its relevant mechanisms during these processes. Finally, the anti-glioma effect and the underlying mechanisms were further confirmed in nude mice bearing U251 tumor xenografts.

## 2. Results

### 2.1. β-Asarone Inhibited the Growth of U251 Cells

**Figure 1:**
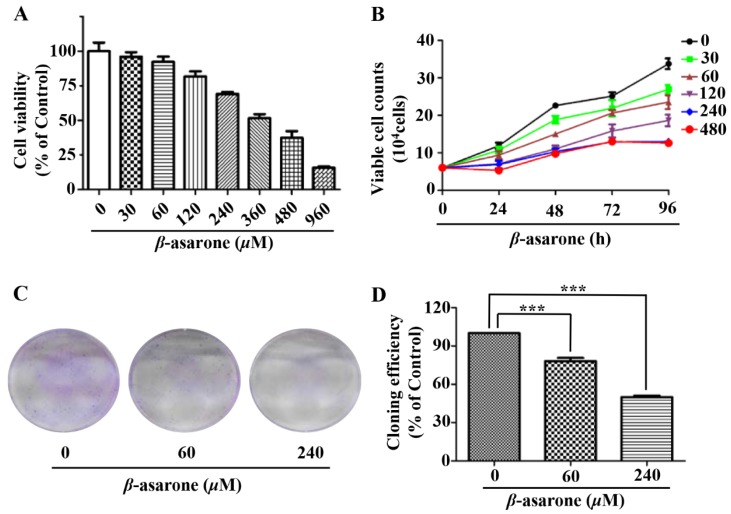
β-asarone inhibited the growth of human glioma U251 cells. (**A**) Cells were treated with β-asarone as indicated for 72 h and the cell viability was determined by SRB assay. (**B**) Cells were treated with β-asarone as indicated for 96 h and cell proliferation was measured by trypan blue exclusion assay. (**C**) Colony formation ability was determined in the cells treated with β-asarone (60 and 240 µM) for two weeks and then stained with 0.005% crystal violet. (**D**) Quantification of cloning efficiency. *** *p* < 0.001 compared with vehicle.

To determine the influence of β-asarone on the growth of human glioma cells, we first evaluated the inhibitory effect of β-asarone on the cell viability of human glioma U251 cells by sulforhodamine B (SRB) assay. Figure 1A demonstrated that β-asarone obviously inhibited the cell viability of U251 cells in a concentration-dependent manner (IC50 = 361 µM). Then, the trypan blue exclusion assay was performed to determine the cell proliferation. Our results showed that β-asarone suppressed the proliferation of U251 cells in a concentration- and time-dependent manner (Figure 1B). Furthermore, the clonogenic assay performed with a sustained treatment of U251 cells with β-asarone for two weeks also indicated that 60 and 240 µM of β-asarone reduced 21.83% and 50.09% of colony formation rate compared with that of the untreated control, respectively (Figure 1C).

### 2.2. β-Asarone Induced Apoptosis of U251 Cells

**Figure 2:**
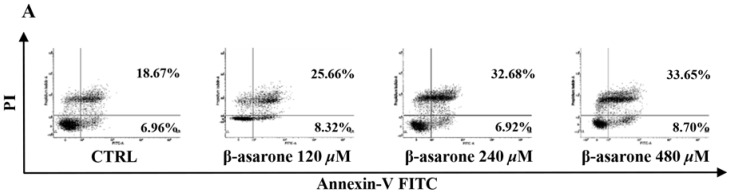

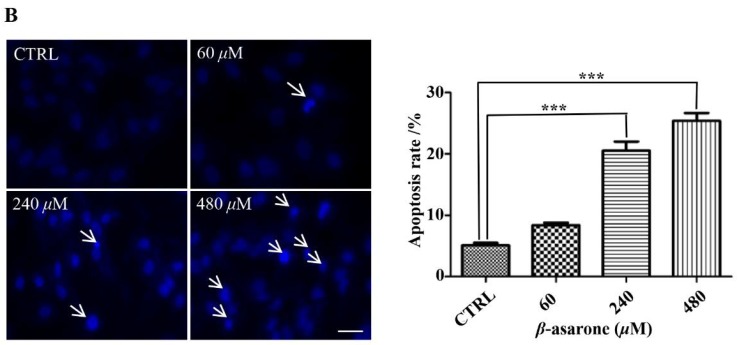
β-asarone induced apoptosis in U251 cells after 48 h treatment. (**A**) Cell apoptosis was determined by flow cytometry after cells were treated with β-asarone as indicated and stained with FITC-conjugated Annexin V and PI. (**B**) Morphologic change of apoptotic cells was detected after cells were treated with β-asarone as indicated and stained with Hoechst 33342. The scar bar is 50 μm. *** *p* < 0.001 compared with vehicle.

To reveal the underlying mechanisms responsible for the β-asarone-mediated growth inhibition of U251 cells, cell apoptosis was first characterized by flow cytometry with Annexin V/PI staining. As shown in Figure 2A, β-asarone (240 and 480 µM) obviously enhanced the apoptosis rate of U251 cells after 48 h treatment. Furthermore, Hoechst 33342 staining was applied to detect the morphologic change of apoptotic cells in U251 cells. Figure 2B showed that U251 cells treated by 240 and 480 µM exhibited much more cells with condensed and fragmented nuclei than control (*p* < 0.001).

### 2.3. β-Asarone Induced Cell Cycle Arrest of U251 Cells

**Figure 3:**
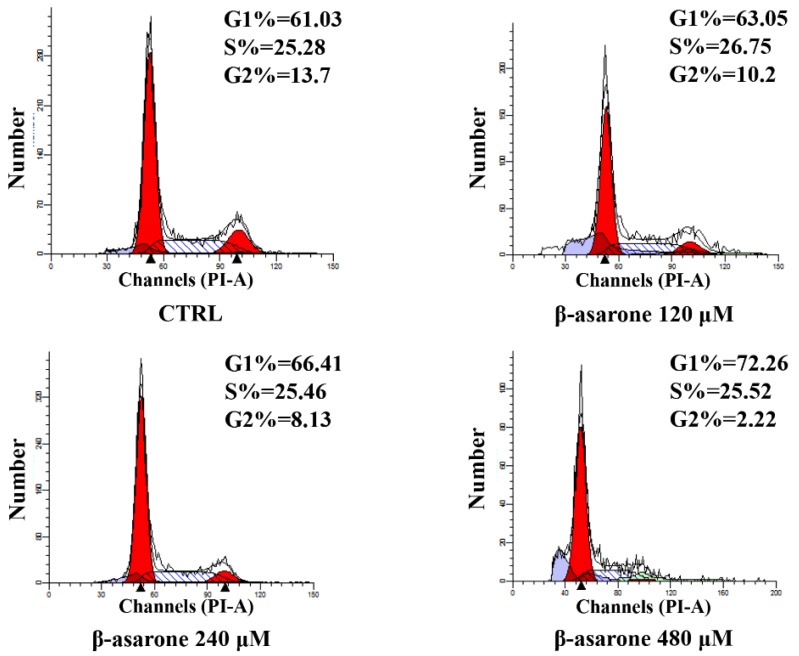
Influence of β-asarone on cell cycle arrest of U251 cells determined by flow cytometry. Cells were treated with β-asarone as indicated for 48 h and stained with PI. The cell cycle distribution was determined by flow cytometry.

To determine whether cell cycle arrest is involved in the β-asarone-mediated growth inhibition of U251 cells, the cell cycle distribution was also determined in our study by flow cytometry with PI staining. As shown in Figure 3, the G1 phase increased from 61.03% to 72.26% after U251 cells were treated with β-asarone from 0 to 480 µM, showing a concentration-dependent manner. Meanwhile, the G2 phase decreased from 13.7% to 2.22% in U251 cells treated by β-asarone (0–480 µM). A G1 cell cycle arrest was caused by β-asarone in U251 cells after 48 h treatment.

### 2.4. Suppression of HnRNP A2/B1 Contributes to β-Asarone-Induced Apoptosis

**Figure 4:**
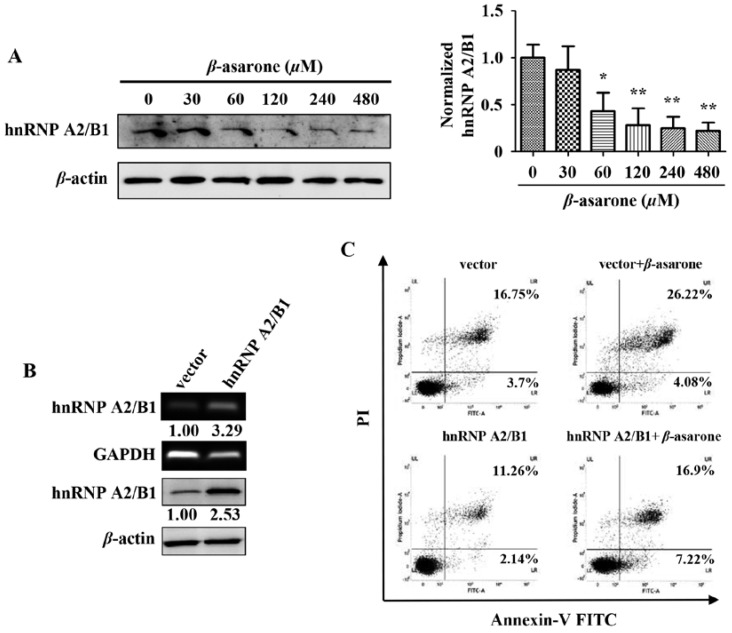
Suppression of hnRNP A2/B1 plays a critical role in β-asarone-induced apoptosis of U251 cells. (**A**) Cells were treated with β-asarone with different concentrations for 24 h. The expression of hnRNP A2/B1 was determined by Western blotting. (**B**) The level of hnRNP A2/B1 was measured by RT-PCR and Western blotting after U251 cells were transfected with pCMV3 or pCMV3-hnRNP A2/B1 for 48 h. The blots were a representative of three independent experiments. (**C**) After being transfected with pCMV3 or pCMV3- hnRNP A2/B1 for 24 h, cells were treated with β-asarone (360 μM) or vehicle for 48 h. The apoptosis rate was evaluated by flow cytometry. * *p* < 0.05 and ** *p* < 0.01 compared with control.

Our previous study with the technique of proteomics revealed that β-asarone decreased the expression level of hnRNP A2/B1 [24], which is a splicing factor and plays a pivotal role in the growth, survival, and invasion of glioma cells [5,11]. Thus, it is interesting for us to explore the potential role of hnRNP A2/B1 in β-asarone-mediated growth inhibition of U251 cells in our following study. Firstly, we further confirmed the effect of β-asarone on hnRNP A2/B1. Our results showed that β-asarone (60–480 μM) concentration-dependently inhibited the protein expression of hnRNP A2/B1 in U251 cells (Figure 4A). Then, we transfected U251 cells with vector or pCMV3-hnRNP A2/B1 to determine whether an overexpression phenotype of hnRNP A2/B1 was established in U251 cells. Figure 4B showed that both mRNA and protein of hnRNP A2/B1 overexpression was observed in U251 cells with pCMV3-hnRNP A2/B1 transfection compared to that with vector transfection. To determine the role of hnRNP A2/B1 in the apoptosis induced by β-asarone, U251 cells were first transfected with vector or pCMV3-hnRNP A2/B1, followed by the treatment with vehicle or 360 μM of β-asarone, which nearly equals to IC50 of β-asarone and also falls into the range of 120 to 480 μM used in Figure 2A and 60 to 480 μM in Figure 4A. Our flow cytometry analysis demonstrated that the apoptosis rate induced by β-asarone obviously reduced after overexpression of hnRNP A2/B1 (Figure 4C).

### 2.5. β-Asarone Promoted the Splicing of Bcl-x via the Inhibition of HnRNP A2/B1

**Figure 5:**
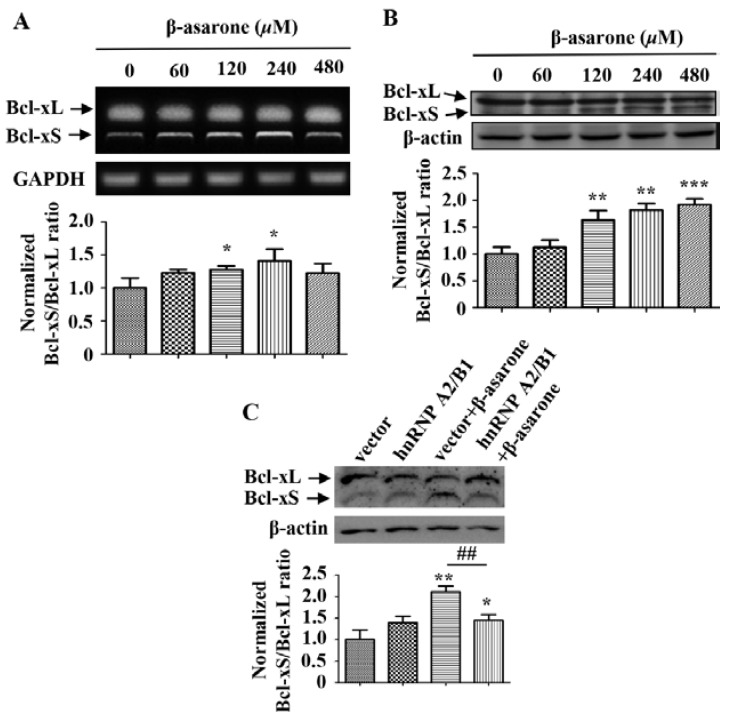
β-asarone enhanced the splicing of Bcl-x through the suppression of hnRNP A2/B1 in U251 cells. (**A**) Cells were treated with β-asarone as indicated for 48 h and the level of Bcl-xL and Bcl-xS was determined by RT-PCR (**A**) and Western blotting (**B**). (**C**) After being transfected with pCMV3 or pCMV3- hnRNP A2/B1 for 24 h, cells were treated with β-asarone (360 μM) or vehicle for 48 h. Then, the level of Bcl-xL and Bcl-xS was determined by Western blotting. The blots were representative of three independent experiments. * *p* < 0.05, ** *p* < 0.01 and *** *p* < 0.001 compared with control. ^##^ *p* < 0.01.

It has been shown that hnRNP A2/B1 plays a critical role in the regulation of apoptosis by participating in the alternative splicing of Bcl-x, which will produce the antiapoptotic Bcl-xL or the proapoptotic Bcl-xS [13,14,15]. Thus, we further determined the influence of β-asarone on the alternative splicing of Bcl-x and the potential role of hnRNP A2/B1. First, we detected the mRNA level of Bcl-xL and Bcl-xS by treating U251 cells with β-asarone of different concentrations. As shown in Figure 5A, β-asarone (120 and 240 μM) obviously changed the alternative splicing of Bcl-x and the ratio of Bcl-xS/Bcl-xL increased with the concentration of β-asarone (*p* < 0.05). Meanwhile, our Western blotting results also demonstrated that β-asarone (120–480 μM) promoted the alternative splicing of Bcl-x (Figure 5B). Then, we evaluated the role of hnRNP A2/B1 in the β-asarone-mediated alternative splicing of Bcl-x. Figure 5C indicated that hnRNP A2/B1 overexpression significantly mitigated β-asarone-mediated alternative splicing of Bcl-x by reducing the ratio of Bcl-xS/Bcl-xL (*p* < 0.01).

### 2.6. Cell Death Receptor Pathway and Mitochondrial Pathway Are Involved in the β-Asarone-Induced Apoptosis of U251 Cells

**Figure 6:**
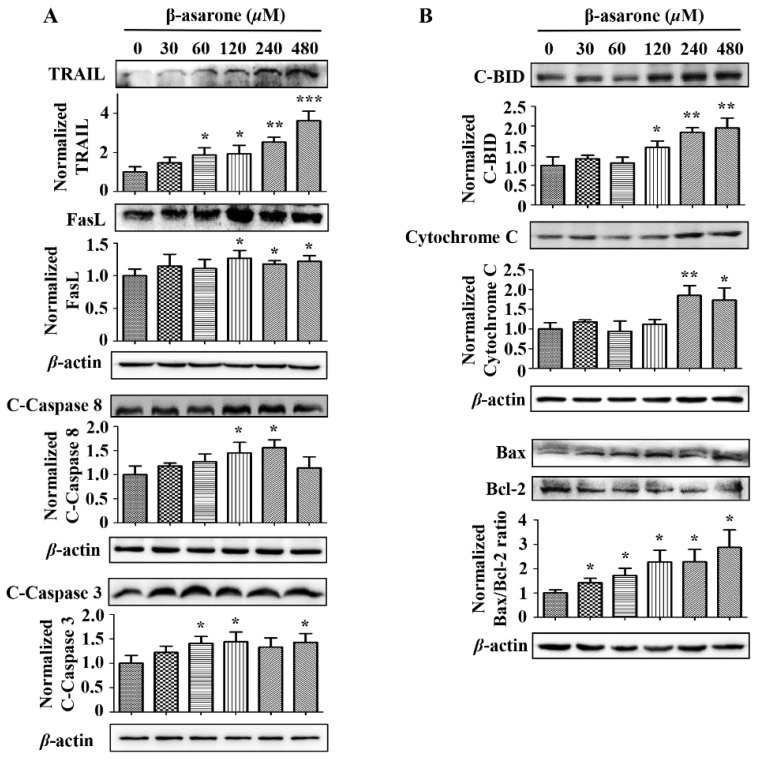
β-asarone modulated the apoptosis-related proteins in U251 cells. Cells were treated with β-asarone as indicated for 48 h and then the key proteins in cell death receptor pathway (**A**) and mitochondrial pathway (**B**) were determined by Western blotting. The blots were a representative of three independent experiments. * *p* < 0.05, ** *p* < 0.01 and *** *p* < 0.001 compared with control.

To further determine the potential signaling pathway involved in the β-asarone-induced apoptosis of U251 cells, several apoptosis-related proteins were determined by Western blotting. Figure 6 revealed that β-asarone significantly promoted the activation of the death receptor proteins TRAIL (60–480 μM of β-asarone) and FasL (120–480 μM of β-asarone), as well as the cleavage of caspase 8 (120 and 240 μM of β-asarone) and caspase 3 (60, 120 and 480 μM of β-asarone) in U251 cells. Moreover, β-asarone (120–480 μM) also obviously enhanced the expression of cleaved-BID, which serves as a critical integrating factor of the death receptor and mitochondrial pathway. Meanwhile, the ratio of Bax/Bcl-2 also significantly increased in U251 cells after β-asarone (30–480 μM) treatment. Additionally, β-asarone (240–480 μM) also enhanced the level of Cytochrome C in the total cellular protein. These data indicate that both the cell death receptor pathway and the mitochondrial pathway may contribute to β-asarone-induced apoptosis of U251 cells.

### 2.7. Suppression of HnRNP A2/B1 Contributes to β-Asarone-Induced Cell Cycle Arrest

**Figure 7:**
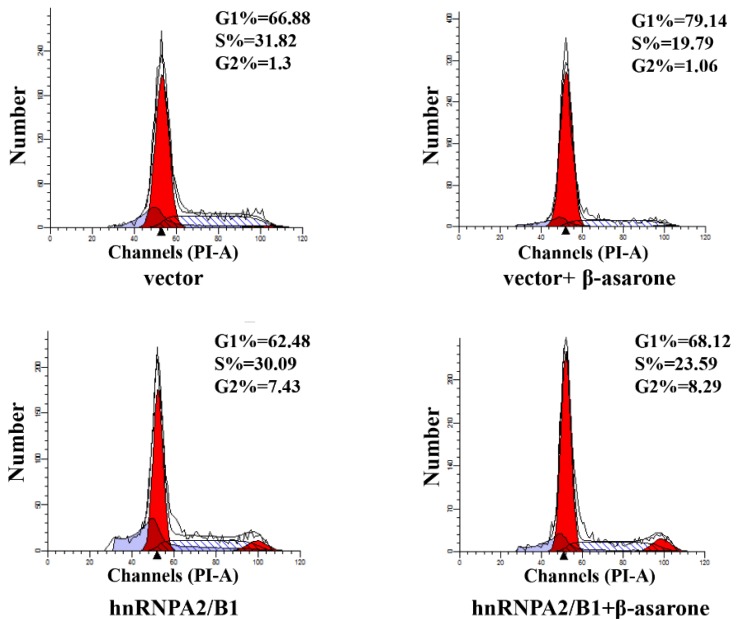
hnRNP A2/B1 overexpression reversed the G1 phase cell cycle arrest caused by β-asarone in U251 cells. After being transfected with pCMV3 or pCMV3-hnRNP A2/B1 for 24 h, cells were treated with β-asarone (360 μM) or vehicle for 48 h. The cell cycle distribution was evaluated by flow cytometry.

Recent investigations have revealed that suppression of hnRNP A2/B1 induced the cell cycle arrest of various cancer cells [17,18,30], indicating the over-expression of hnRNP A2/B1 in tumor cells is highly correlated to the uncontrolled cell cycle. To further determine the potential role of hnRNP A2/B1 in β-asarone-induced cell cycle arrest of U251 cells, we first transfected U251 cells with vector or pCMV3-hnRNP A2/B1 and then treated U251 cells with vehicle or β-asarone. The flow cytometry analysis (Figure 7) demonstrated that β-asarone caused cell cycle arrest at G1 phase (from 66.88% to 79.14%) in U251 cells transfected with vector only. However, overexpression of hnRNP A2/B1 obviously reversed the G1 phase cell cycle arrest (from 79.14% to 68.12%) induced by β-asarone in U251 cells transfected with pCMV3-hnRNP A2/B1.

### 2.8. β-Asarone Modulated the Expression of Cell Cycle Related Proteins in U251 Cells

**Figure 8:**
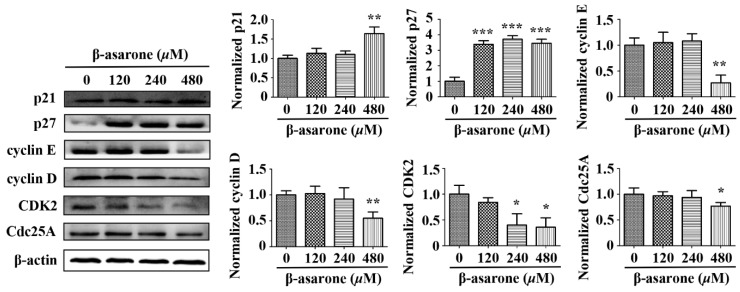
Influence of β-asarone on cell cycle related proteins of U251 cells (*n* = 3). Cells were treated with β-asarone as indicated for 48 h and the proteins related to cell cycle were determined by Western blotting. The blots were representative of three independent experiments. * *p* < 0.05, ** *p* < 0.01 and *** *p* < 0.001 compared with control.

In normal cells, the cell cycle is strictly regulated by cell cycle related proteins, including cyclin-dependent kinases (CDKs), cyclins and cyclin-dependent kinase inhibitors (CKIs), while those proteins usually exhibit abnormal expression in tumor cells, leading to the dysfunction of cell cycle checkpoint [31,32]. Thus, we determined the potential influence of β-asarone on the expression of p21 and p27 (CKIs), cyclin D and cyclin E (cyclins), CDK2 (CDKs) and Cdc25A (the activator of CDKs), which were described associated with G1 phase cell cycle arrest and hnRNP A2/B1 mediated cell cycle dysregulation [16,17,18,19]. As shown in Figure 8, Western blotting analysis demonstrated that p21 in U251 cells increased with β-asarone (480 μM) treatment, while p27 increased with β-asarone (120–480 μM) treatment. Moreover, cyclin D, cyclin E and Cdc25A decreased with β-asarone (480 μM) treatment, while CDK2 decreased with β-asarone (240 and 480 μM) treatment.

### 2.9. β-Asarone Suppressed the Tumor Growth of U251 Xenografts in Nude Mice

**Figure 9:**
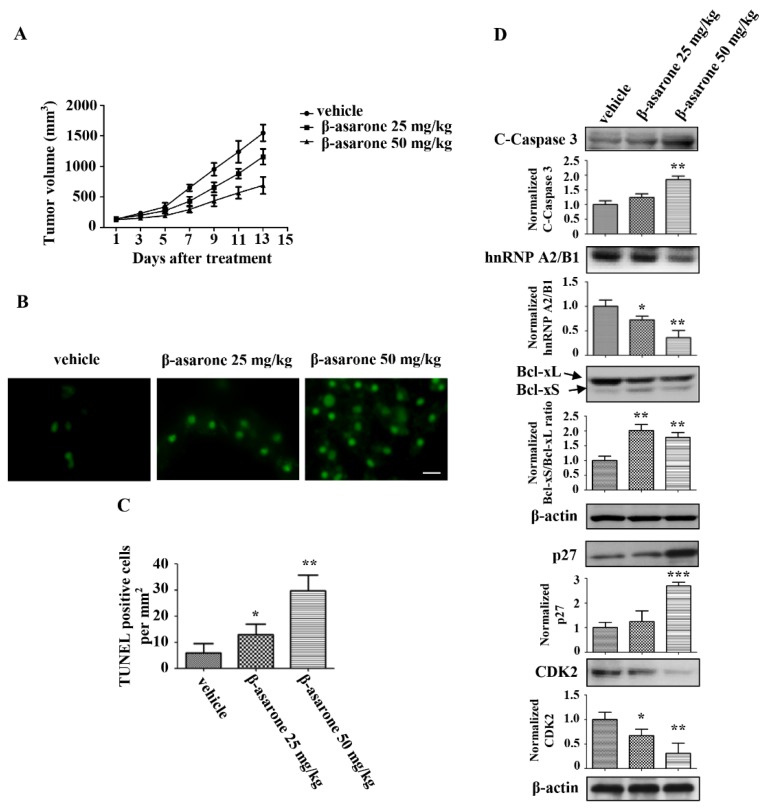
β-asarone inhibited U251 xenograft tumor growth in nude mice. (**A**) The tumor models were established in nude mice (six/group) with the injection of U251 cells into the right flank of the mice subcutaneously and then treated with vehicle or β-asarone (25 and 50 mg/kg) p.o. daily for one week. (**A**) Tumor volume growth curve. Tumor sizes were measured every other day. (**B**) Apoptosis of xenograft tissues in different groups were evaluated by TUNEL assay. The scar bar is 50 μm. (**C**) Quantification of TUNEL assay. (**D**) The expression of cleaved-caspase 3, Bcl-xL, Bcl-xS and hnRNP A2/B1 in tumor tissues of different groups was determined by Western blotting. The blots were a representative of three independent experiments. * *p* < 0.05, ** *p* < 0.01 and *** *p* < 0.001 compared with vehicle.

The anti-tumor activity of β-asarone was further investigated in vivo using nude mice with U251 xenografts. As shown in Figure 9A, tumor growth of the U251 xenografts was obviously suppressed following treatment with β-asarone (25 and 50 mg/kg) compared with that treated with vehicle, with 25.2% and 55.5% reduction for 25 and 50 mg/kg of β-asarone, respectively. The TUNEL assay with the isolated tumors revealed that the apoptotic rate in the tumors was significantly enhanced by β-asarone (25 and 50 mg/kg) compared with that in vehicle group (Figure 9B,C). Furthermore, we analyzed the expression of apoptotic proteins in tumor tissues by Western blotting. Figure 9D shows that an increase in cleaved caspase-3 was observed in tumor tissue obtained from β-asarone (50 mg/kg) group compared with that obtained from vehicle group (*p* < 0.05). More importantly, the expression of hnRNP A2/B1 dose-dependently decreased in the β-asarone (25 and 50 mg/kg) groups compared with those in the vehicle group. Meanwhile, the ratio of Bcl-xS/Bcl-xL also significantly increased in the β-asarone (25 and 50 mg/kg) groups compared with those in the vehicle group (*p* < 0.01). Moreover, the expression of p27 increased in the β-asarone (50 mg/kg) groups, while the expression of CKD2 decreased in the β-asarone (25 and 50 mg/kg) groups.

## 3. Discussion

*Acori tatarinowii* Rhizoma has been used as a traditional herbal medicine in China for central nervous system (CNS) disorders. Recently, we found that the volatile oil of Acori tatarinowii Rhizoma induced apoptotic cell death and protective autophagy in human glioma cells [20]. Furthermore, its main component β-asarone was found to inhibit the cell viability of human glioma cells in our subsequent investigation. Meanwhile, hnRNP A2/B1, a well characterized splicing factor, was found to be suppressed by β-asarone in our proteomic study [24]. Here, we present evidence that hnRNP A2/B1-mediated signaling pathway may contribute to β-asarone-induced growth inhibition of human glioma cells.

Recently, hnRNP A2/B1 has drawn increasing attention due to the overexpression in a number of tumors, including human glioma tissues [5,6,7,8,9,10,11]. Moreover, emerging evidence has firmly established that that the level of hnRNP A2/B1 is highly associated with a more aggressive tumor phenotype and poor prognosis. In human glioblastoma, hnRNP A2/B1 was found to be an oncogenic driver and strongly related to the invasion, growth and survival of tumor cells [5,11]. Thus, targeting hnRNP A2/B1 may provide potential approaches for the therapeutic intervention of glioma. Interestingly, hnRNPA2/B1 has been identified in our recent study as one of the key protein targets regulated by β-asarone in U251 cells with a two-dimensional gel electrophoresis (2-DE)-based proteomic strategy [24]. In the current study, we further characterized the effect of β-asarone on hnRNPA2/B1 and found that β-asarone suppressed the protein level of hnRNPA2/B1 in a concentration-dependent way. Importantly, overexpression of hnRNPA2/B1 obviously reversed the induction of β-asarone on apoptosis, indicating the critical role of hnRNPA2/B1 in β-asarone-mediated apoptosis.

Alternative splicing plays a central role in the generation of multiple protein isoforms with opposing functions through the splicing in the specific site within the pre-mRNA of the target gene [33]. HnRNPA2/B1 is a well characterized RNA-binding splicing factor and has been shown to regulate the cell proliferation, survival, and invasiveness through the alternative processing of several pre-mRNA targets [5,13,14,15,33,34]. One example is Bcl-x, a member of the well-known Bcl-2 family, that is alternatively spliced as the antiapoptotic Bcl-xL or the proapoptotic Bcl-xS. In tumor cells, hnRNP A2/B1 was found to serve as basic machinery, whereas hnRNP A2/B1 binds to Bcl-x pre-mRNA with the assistance of BC200, an ER-regulated lncRNA, to form a BC200-Bcl-x-hnRNP A2/B1 complex, resulting in the suppression of Bcl-xS expression and the promotion of Bcl-xL expression [13,14,15]. Thus, downregulation of the level or activity of hnRNPA2/B1 would be expected to increase the formation of Bcl-xS and induce apoptosis. Our study demonstrated that the ratio of Bcl-xS/Bcl-xL was enhanced by β-asarone in both mRNA and protein level, suggesting that β-asarone promoted the alternative splicing of Bcl-x. Furthermore, overexpression of hnRNPA2/B1 mitigated the promoting effect of β-asarone on the alternative splicing of Bcl-x in U251 cells. We speculate from the results that blockage of hnRNPA2/B1’s negative effect on alternative splicing of Bcl-x may be critical in β-asarone-apoptosis of U251 cells. Another interesting finding in this study is that both cell death receptor pathway and mitochondrial pathway may be involved in the β-asarone-induced apoptosis of U251 cells. This finding is supported by our Western blotting results that β-asarone activated the signaling proteins TRAIL, FasL, and caspase 8, which are involved in the death receptor pathway and enhance the ratio of Bax/Bcl-2 also and the level of cytochrome C, which are associated with the mitochondrial pathway. 

Recent investigation demonstrated that suppression of hnRNP A2/B1 caused the cell cycle arrest at G1 phase in cervical cancer cells [16], lung cancer cells [17,18] and human embryonic stem cells [19]. In the current study, our flow cytometry analysis indicated that β-asarone induced G1 phase of cell cycle arrest in U251 cells. Thus, it is interesting to us that whether the G1 phase arrest caused by β-asarone is correlated with the inhibitory effect of β-asarone on hnRNP A2/B1. Our subsequent investigation demonstrated that overexpression of hnRNP A2/B1 obviously reversed the G1 phase arrest in U251 cells caused by β-asarone, suggesting that there is a direct correlation between the G1 phase arrest and the inhibition on hnRNP A2/B1 induced by β-asarone. Usually, the cell cycle is tightly regulated by cell cycle related proteins [31,32]. Thus, we then determined the effect of β-asarone on the expression of cell cycle related proteins. Western blotting analysis revealed that β-asarone-induced cell cycle arrest is related to the upregulation of p21 and p27 and inhibition of Cdc25A, cyclin D, cyclin E and CDK2. It is reported that the upregulation of p21 and p27 was observed in the cervical cancer cells with G1 phase arrest caused by the inhibition of hnRNP A2/B1 [16]. Accumulating evidences indicate that cyclin D, cyclin E and Cdc25A are the proteins required by the transition of cell cycle G0/G1 phase, while CDK2 is an important regulation factor during the transition of G1/S phase [31,32]. Previous study revealed that the downregulation of cyclin D, cyclin E, Cdc25A and CDK2 was found in the human embryonic stem cells with G1 phase arrest caused by the suppression of hnRNP A2/B1 [19]. Thus, the G1 phase arrest caused by β-asarone via the suppression of hnRNP A2/B1 may be closely correlated with the upregulation of p21 and p27 and inhibition of Cdc25A, cyclin D, cyclin E and CDK2.

Finally, anti-tumor activity was confirmed in nude mice with U251 xenografts. β-asarone was shown to inhibit the tumor growth and induce the apoptosis of U251 xenografts. Similar effect of β-asarone has also been observed in LoVo colon cancer cells [22]. Notably, we found that β-asarone suppressed the hnRNP A2/B1 expression in U251 xenografts. Moreover, β-asarone enhanced the expression of cleaved-caspase 3 and the ratio of Bcl-xS/Bcl-xL, which are associated with β-asarone-induced apoptosis, and upregulated the expression of p27 and downregulated the expression of CDK2, which are correlated with β-asarone-induced cell cycle arrest.

Taken together, the results obtained in this study demonstrates that β-asarone inhibited the growth and induced apoptosis and cell cycle arrest of U251 cells through the suppression of hnRNP A2/B1-mediated signaling pathway. On the one hand, β-asarone promoted the alternative splicing of Bcl-x, resulting in enhancing the ratio of Bcl-xS/Bcl-xL, by the inhibition of hnRNP A2/B1, which may be correlated with β-asarone-induced apoptosis via mitochondrial pathway. On the other hand, β-asarone induced the G1 phase cell cycle arrest through the suppression of hnRNP A2/B1 and the modulation of p21, p27, Cdc25A, cyclin D, cyclin E and CDK2.

## 4. Materials and Methods

### 4.1. Chemicals and Reagents

Beta-Asarone was purchased from Dalian Meilun Biological Technology Co., Ltd. (Dalian, China) with a purity higher than 98% and dissolved in dimethyl sulfoxide (DMSO) and stored at −20 °C before use. The antibodies against hnRNP A2/B1 were obtained from Cell Signaling Technology (Boston, MA, USA). The antibodies against TRAIL, Fas-L, Cleaved-Caspase 8 (C-Caspase 8), Cleaved-BID (C-BID), Bax, Bcl-2 p21, p27, Cdc25A, cyclin D, cyclin E, CDK2 and β-actin were obtained from Wanlei Biotechnology (Shenyang, China) and diluted with the factor of 1:500. The antibodies against Cytochrome C, Cleaved-Caspase 3 (C-Caspase 3) and Bcl-x were obtained from Santa Cruz Biotechnology (Santa Cruz, CA, USA) and diluted with the factor of 1:1000. Other chemicals were obtained from Sigma-Aldrich (Burlington, MA, USA), unless indicated otherwise indicated.

### 4.2. Cell Culture 

Human glioma U251 cells were obtained from the American Type Cell Culture Collection (Manassas, VA, USA) and maintained in Dulbecco’s modified Eagle’s medium (DMEM) supplemented with 10% fetal bovine serum (FBS) (Invitrogen, Carlsbad, CA, USA), 1% penicillin/streptomycin (Invitrogen) at 37 °C in a 5% CO_2_ and 95% air atmosphere.

### 4.3. Cell Viability Assay

Sulforhodamine B (SRB) assay was used to determine cell viability. After drug treatment, U251 cells were fixed with 50% (*w*/*v*) trichloroacetic acid and incubated at 4 °C for 1 h. Then, the cells were stained with 100 μL of SRB solution for 15 min and the unbound dye was removed by washing with 1% acetic acid. The bound dye was dissolved by 150 μL of 10 mM Tris base (pH 10.5). The OD value was detected at 515 nm with a microplate reader (Biotek, Winooski, VT, USA). Cell viability was presented as a percentage of untreated cells.

### 4.4. Trypan Blue Dye Exclusion Test

The inhibitory effect of β-asarone on U251 cells was determined using trypan blue solution (Sigma, St. Louis, MO, USA). Cells were treated separately for 24, 48, 72, or 96 h and then stained with trypan blue (0.4%). The viable cells were counted using a hemocytometer.

### 4.5. Colony Formation Assay

U251 Cells were seeded in a 6-well plate and pretreated with β-asarone or vehicle for two days and then treated with β-asarone for 14 days. The medium with β-asarone or vehicle was replaced every three days. At the end of treatment, cells were fixed in 100% methanol and stained with 0.005% crystal violet. Finally, images were captured by a SONY camera (Tokyo, Japan) and the colonies were counted.

### 4.6. Cell Apoptosis Analyzed by Flow Cytometry

U251 Cells were seeded and cultured overnight in six-well plates. After treatment, cells were harvested, washed and re-suspended in the binding buffer containing annexin V and propidiumiodide (PI). After incubation at room temperature in the dark for 20 min, the stained cells were subjected to a BD LSRFortessa Cell Analyzer (BD Biosciences, San Jose, CA, USA) with fluorescence emission at 530 nm and 575 nm and excitation at 488 nm. Data were analyzed using Flow Jo 7.6.1 software (Tree Star, Inc., Ashland, OR, USA).

### 4.7. Hoechst 33342 Staining

U251 cells were seeded and cultured overnight in 6-well plate. After treatment, cells were washed with 1 × PBS for 3 times. Then, Hoechst 33342 dissolving in 1 × PBS was added into each well. The plate was kept at room temperature for 10 min and avoided from light. Finally, the plates were washed with 1 × PBS again and images were captured under the fluorescence microscope (Nikon, Tokyo, Japan).

### 4.8. Western Blotting Analysis

The U251 Cells after drug treatment were lysed with ice-cold RIPA buffer (Cell Signaling Technologies, USA) supplemented with 1% (*v*/*v*) protein inhibitor cocktail and 1 mM phenylmethylsulfonyl fluoride (PMSF). Then, proteins were separated by electrophoresis in 10–12.5% SDS-polyacrylamide gel, and subsequently transferred to polyvinylidene difluoride (PVDF) membrane. After blocked with 5% nonfat milk for 1 h at room temperature, the membranes were incubated with respective primary antibodies at 4 °C overnight. Following this, the membranes were washed, and incubated with horseradish peroxidase (HRP)-conjugated secondary antibodies at room temperature for 1 h. The blots were visualized by enhanced chemiluminescence (ECL) detection reagents (GE Healthcare, Uppsala, Sweden). The blots were acquired and quantified by a gel imaging system (Tanon, Shanghai, China). The concentration of the loaded cellular proteins was normalized against the internal control β-actin, and then the value was expressed as each normalized data relative to control.

### 4.9. Cell Cycle Distribution Analyzed by Flow Cytometry

After treatment, cells were harvested, washed twice with ice-cold PBS (pH 7.4), and fixed in 70% ethanol overnight at 4 °C. Then, cells were incubated with 250 μL of RNase A (100 μg/mL) for 30 min at 37 °C and finally stained with 500 μL of PI (50 μg/mL) for 1 h in the dark. Stained cells were analyzed with a BD LSRFortessa Cell Analyzer (BD Biosciences, San Jose, CA, USA). Data were analyzed using Flow Jo 7.6.1 software (Tree Star, Inc., Ashland, OR, USA).

### 4.10. Plasmids and Transient Transfection

The pCMV3-hnRNPA2/B1 and pCMV3 vectors were purchased from Sino Biological Inc. (Beijing, China). Cells (50% confluence) were transfected with 2.5 μg of DNA using the transfection reagent Lipofectamine 2000 (Invitrogen, USA) following the protocol provided by the manufacturer. Transfected cells were first cultured in antibiotic-free medium for 6 h and then in fresh medium for 48 h, followed by further drug treatments.

### 4.11. Semi-Quantitative RT-PCR Detection

Total RNA was extracted from U251 cells by using TriZol RNA extraction reagent (Invitrogen). Single-strand cDNA was synthesized using SuperScript^®^ III reverse transcription reagent (Invitrogen) according to the manufacturer’s protocol. hnRNP A2/B1 was detected using the forward primer and the reverse primer 5’-GCAACCTTCTAACTACGGTCCA-3’, 5’-ATCTGCTCTGGTGTCTTCTGC-3’, Bcl-x was detected using the forward primer 5’-GGGTCTAGAAGTGGATGGTCAGTGTCTGGT-3’ and the reverse primer 5’-GGGGAATTCTTGGACAATGGACTGGTTGA-3’, where GAPDH was detected using the forward primer 5’-TGTTGCCATCAATGACCCCTT-3’ and the reverse primer 5’-CTCCACGACGTACTCAGCG-3’. Both Bcl-xL (779 bp) and Bcl-xS (590 bp) were amplified from the Bcl-x primers. PCR amplification was set as follows: after an initial denaturation at 94 °C for 2 min, 30 cycles of 94 °C for 30 s, 55 °C (hnRNP A2/B1)/63 °C (Bcl-x)/55 °C (GAPDH) for 30 s, and 72 °C for 60 s. The reaction ended with a final extension step at 72 °C for 5 min. PCR products were separated by gel electrophoresis in 1.2% agarose containing ethidium bromide and visualized under UV light.

### 4.12. Nude Mouse Xenograft Model

Nude mice (Charles River, Beijing, China), aged 4–6 weeks (~20 g body weight), were used to test the in vivo effects of β-asarone. U251 cells (5 × 10^7^) were injected into the right flank of the mice subcutaneously. Animals were treated with different doses of β-asarone, including 25 mg/kg, and 50 mg/kg and vehicle (0.9% saline containing 1.25% tween-80) p.o. daily for one week. When the treatment began, the tumor volume was measured by caliper and calculated using the formula: tumor volume = (length × width^2^)/2. Growth curves were plotted using average tumor size within each experimental group at the defined time points. When the tumor size of the control group reached the IACUC endpoint, the animals were sacrificed, and the tumors were harvested for TUNEL staining and Western blotting. All experiments were performed under the supervision of the Institutional Animal Care and Use Committee of Southwest University according to an approved protocol.

### 4.13. Terminal Dexynucleotidyl Transferase-Mediated dUTP Nick End Labeling (TUNEL) Assay

Apoptosis in tumor tissue was determined with a standard in situ TUNEL method following the manufacturer’s directions (Promega, Madison, WI, USA). Images of the sections were taken by a fluorescence microscope (Olympus, Tokyo, Japan) and images were analyzed by a person who was blinded to the animal group information. Apoptotic cells were counted as the number of TUNEL positive cells per mm^2^ of each examined section.

### 4.14. Statistical Analysis

All data were presented as mean ± SD for three independent experiments. Statistical analysis was performed by a two-tailed Student’s *t*-test or one-way analysis of variance (ANOVA). A *p*-value of less than 0.05 was considered to be statistically significant.

## Abbreviations

hnRNPs	Heterogeneous nuclear ribonucleoproteins
BBB	blood–brain barrier
SRB	Sulforhodamine B
DMSO	dimethyl sulfoxide
TUNEL	Terminal Dexynucleotidyl Transferase-Mediated dUTP Nick End Labeling
C-Caspase 8	Cleaved-Caspase 8
C-BID	Cleaved-BID
C-Caspase 3	Cleaved-Caspase 3
CDKs	cyclin-dependent kinases
CKIs	cyclins and cyclin-dependent kinase inhibitors
